# Left atrial appendage as a vantage point for mapping and ablating premature ventricular contractions originating in the epicardial left ventricular summit

**DOI:** 10.1002/ccr3.1525

**Published:** 2018-04-25

**Authors:** Daniel Benhayon, John Cogan, Ming Young

**Affiliations:** ^1^ Memorial Healthcare System Hollywood Florida

**Keywords:** Catheter ablation, left atrial appendage, left ventricular summit, ventricular tachycardia

## Abstract

Idiopathic ventricular tachycardia arising from the LV summit epicardial area can be successfully mapped and possibly ablated from the left atrial appendage.

## Introduction

The left ventricular (LV) summit is a common site from where idiopathic premature ventricular contraction (PVC) and ventricular tachycardia (VT) can originate.

Catheter ablation of the LV summit can pose various anatomical challenges, and foci located deep in the myocardium often need more extensive ablation to surround the area from where it originates, in order to be successful.

We present a case of idiopathic VT arising from the LV summit area, where the left atrial appendage was used as a vantage point to successfully map and ablate the epicardial aspect of the septal LV/LV summit area. This approach can supplement the epicardial mapping of the LV summit currently performed via the great cardiac vein (GCV) and the anterior interventricular vein (AIV), or replace it when the veins are not approachable due to anatomical challenges.

## Case Report

A 51‐year‐old man with no significant past medical history was referred to our institution with complaints of frequent palpitations and fast palpitations with near syncope associated with exercise. He was being treated with metoprolol succinate. There was no family history of sudden death. As part of his workup, an ECG showed a normal QRS in sinus rhythm, with evidence of occasional PVCs with a left bundle, right inferior axis (Image 1); an echocardiogram revealed a normal left and right ventricular function with normal valve function. While undergoing a treadmill stress test, at stage 3 of the Bruce protocol, he developed fast monomorphic VT matching his PVC morphology and associated with near syncope. A cardiac MRI corroborated a normal LV and RV function, and it was negative for any late gadolinium enhancement. Coronary angiography was negative for obstructive coronary disease.

At that point, the patient was brought to the electrophysiology laboratory for EP study and ventricular tachycardia ablation. Under light sedation, a Josephson quad catheter was placed in the right ventricular apex, an 8F SoundStar intracardiac echocardiography (ICE) catheter was advanced into the heart to reconstruct the cardiac anatomy using the Carto‐Sound module (Biosense Webster, Diamond Bar, CA) and to assist with catheter movement and mapping. An 8F ThermoCool^®^ Smart Touch SF (Biosense Webster, Diamond Bar, CA)‐irrigated tip catheter was then advanced into the right ventricle through an Agilis steerable sheath (Abbott/St. Jude Medical, St. Paul, MN). The Carto‐3 3D mapping system (Biosense Webster) was used for mapping. Activation mapping localized the earliest site of activation at the anterior‐most leftward aspect of the RV outflow tract, about 2 cm under the pulmonic valve in what is also known as the site 6 RVOT (−24 msec pre‐QRS). Pace‐mapping in this area yielded an almost perfect match at >97% using the PASO module (Biosense Webster).

**Figure 1:**
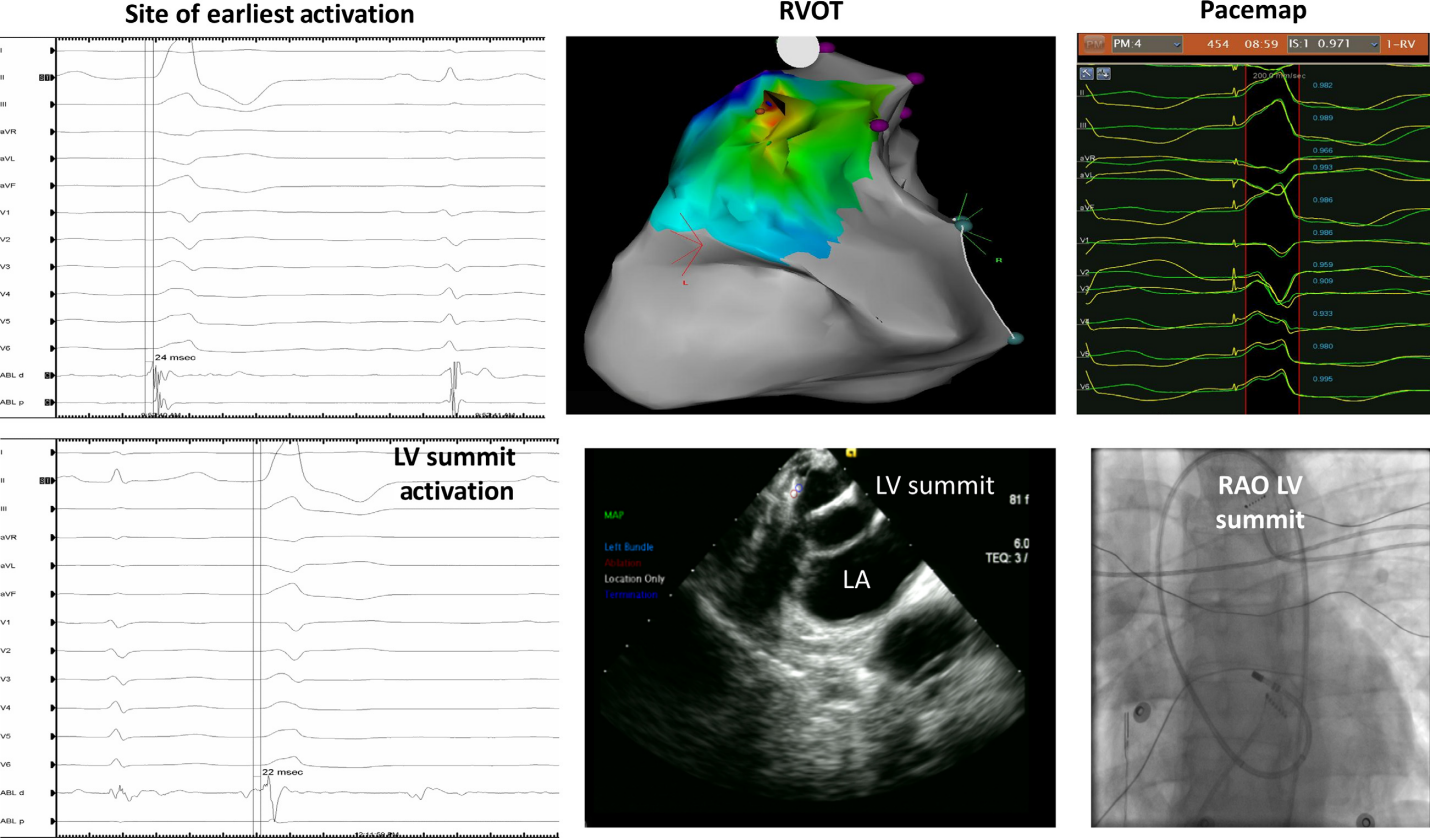
Site of earliest activation in the RVOT and the LV summit area. From left to right, the signal through our recording system, the electro‐anatomical map showing the area of earliest activation and the corresponding pacemap.

After heparin was infused to achieve an ACT >250 sec, the ablation catheter was advanced into the coronary cusp region and LV via a retrograde approach for mapping. The earliest activation was noted at the endocardial aspect of the LV summit, under the R/L junction of the coronary cusps (−20 msec pre‐QRS); pace‐mapping yielded a poor match (Fig. 1).

The GCV/AIV was attempted to be reached via the coronary sinus, but it became small in its distal portion and the ablation catheter could not be advanced all the way to reach the epicardial aspect of the LV summit.

At that point, RF was delivered at the site of earliest activation in the RVOT region (20–30 Watts for 60–90 sec). The PVCs would suppress during the ablation but then come back at a similar burden after energy delivery. On ICE evaluation, it was noted that the structure adjacent to where we were ablating was the tip of the left atrial appendage.

**Figure 2:**
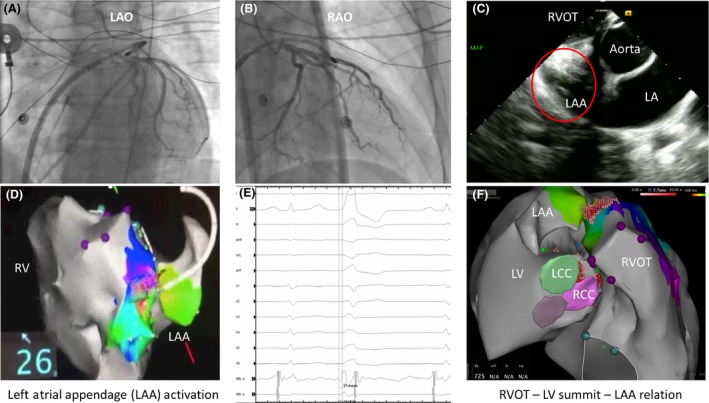
Mapping of the LV summit PVC using the left atrial appendage. A and B show the fluoroscopy projections with the catheter placed inside the appendage resting over the epicardial LV. The relationship of the appendage and the coronary arteries is shown in the LAO and RAO projection. (C) shows the ICE image with the catheter at the tip of the left atrial appendage. The ICE probe is positioned inside the RVOT and tilted up to visualize the left atrial appendage. (D) Electro‐anatomical map showing the left atrial appendage and the RVOT relationship. (E) The PVC signal recorded through the appendage. (F) Carto‐Sound reconstruction of the anatomy showing the intimate relation of the RVOT, LVOT, and left atrial appendage.

Now with ACTs reaching >300 sec and under ICE and fluoroscopic guidance, a single transseptal was performed and the Agilis steerable sheath was advanced into the left atrium. The ablation catheter was then advanced into the tip of the left atrial appendage and with the vector pointed toward the LV, the PVC was mapped. The earliest activation was noted in this area, although the signal was of a far‐field nature (−31 pre‐QRS), pacing in this area would capture the atrial tissue and not the ventricular tissue (Fig. 2).

After a coronary angiogram was performed to make sure we were not in close proximity to any major coronary vessels, a lesion was delivered (20–30 Watts for 40 sec), and again, the PVC foci were partially suppressed but not eliminated.

Further ablation on the RVOT, left atrial appendage, and the LV endocardium, with the goal of surrounding the foci, which at this point seemed to be located in the intramyocardial aspect of the LV summit, successfully eliminated it. Postablation ventricular burst pacing on–off isoproterenol could not re‐induce the VT or PVC, and the subsequent Holter testing and treadmill testing have confirmed the long‐term elimination of the ectopy. There were no complications.

### Comments

The LV summit as it is known in the anatomy literature is the highest point of the left ventricle, anterior to the aortic valve, and represents a common site of origin for idiopathic outflow tract VT or PVCs. The area can be approached using various structures as vantage points. The common sites are the left and right coronary cusp, its endocardial aspect by advancing the catheter into the LV, the great cardiac vein and interventricular vein, the anterior‐most leftward aspect of the RVOT, and lately using unipolar wires that can penetrate the venous septal perforators 1, 2, 3.

In the present case, we describe the use of the left atrial appendage as a structure that can also be used as a vantage point to reach the LV summit. The left atrial appendage has many anatomical variants, but it often rests on the lateral aspect of the LV and its tip can span all the way to reach the epicardial LV summit and even touch the RVOT, where the two ventricles meet 4.

In this case, we could not approach the GCV or AIV to use it to map the epicardial septal aspect of the LVOT, and often, the ability to deliver energy at that site is hampered by the course of the left anterior descending coronary artery.

We believe that the left atrial appendage can be utilized to map this region and gives us another option. The left atrial appendage has multiple bundles of muscles separated by areas that can be paper thin 4, and underneath it, there can be some fat tissue that may prevent adequate energy delivery to the ventricular muscle. The partial suppression noted in our case may suggest its feasibility; furthermore, assuming that the point of maximum temperature when using irrigated catheters is about 5 mm from the catheter surface, it is theoretically possible to assume that some energy is effectively reaching the epicardial muscle. Nevertheless, animal studies are needed to further explore the point and to establish if it would be safe in most cases.

Limitations associated with the course of the great epicardial coronary arteries are also relevant when ablating in this structure, but we can safely establish that when the anatomy is favorable, the left atrial appendage can be used as a structure from where the epicardial aspect of the LV summit could be mapped.

## Authorship

DB: performed the case and the concept of utilizing the left atrial appendage to map the arrhythmia in this challenging case, in conjunction with Dr John Cogan at our institution, wrote, and revised the manuscript with both authors. MY: is the most senior member of our group and revised the present manuscript and tracings involved in the figures.

## Conflict of Interest

There are no conflict of interests for any of the authors to disclose.

## References

[1] Yamada, T. , H. T. McElderry , H. Doppalapudi , Y. Murakami , Y. Yoshida , N. Yoshida , et al. 2008 Idiopathic ventricular arrhythmias originating from the aortic root: prevalence, electrocardiographic and electrophysiologic characteristics, and results of radiofrequency catheter ablation. J. Am. Coll. Cardiol. 52:139–147. 18598894 10.1016/j.jacc.2008.03.040

[2] Yokokawa, M. , E. Good , A. Chugh , F. Jr Pelosi , T. Crawford , K. Jongnarangsin , et al. 2012 Intramural idiopathic ventricular arrhythmias originating in the intraventricular septum: mapping and ablation. Circ. Arrhythm. Electrophysiol. 5:258–263. 22407415 10.1161/CIRCEP.111.967257

[3] Yamada, T. , S. H. Litovsky , and G. N. Kay . 2008 The left ventricular ostium: an anatomic concept relevant to idiopathic ventricular arrhythmias. Circ. Arrhythm. Electrophysiol. 1:396–404. 19808434 10.1161/CIRCEP.108.795948

[4] Ho, S. Y. , J. A. Cabrera , and D. Sanchez‐Quintan . 2012 Left atrial anatomy revisited. Circ. Arrhythm. Electrophysiol. 5:220–228. 22334429 10.1161/CIRCEP.111.962720

